# Adaptive Cuckoo Search Algorithm for Unconstrained Optimization

**DOI:** 10.1155/2014/943403

**Published:** 2014-09-14

**Authors:** Pauline Ong

**Affiliations:** Faculty of Mechanical and Manufacturing Engineering, Universiti Tun Hussein Onn Malaysia (UTHM), 86400 Parit Raja, Batu Pahat, Johor, Malaysia

## Abstract

Modification of the intensification and diversification approaches in the recently developed cuckoo search algorithm (CSA) is performed. The alteration involves the implementation of adaptive step size adjustment strategy, and thus enabling faster convergence to the global optimal solutions. The feasibility of the proposed algorithm is validated against benchmark optimization functions, where the obtained results demonstrate a marked improvement over the standard CSA, in all the cases.

## 1. Introduction

The solutions to multitudinous domains-whether in engineering design, operational research, industrial process, or economics inevitably have optimization at heart. However, having a solid grasp for such problems turns out to be painstakingly tough and tedious; and thus, gearing towards an efficient and effective algorithm in the light of solving increasingly complex optimization problems in practice is of paramount significance. Extensive and intensive studies in this aspect fruit in numerous optimization techniques, particularly, the bioinspired metaheuristic methods which draw inspiration from the means on how humans and living creatures struggle to survive in a challenging environment, for instance, genetic algorithm (GA) [1], particle swarm optimization (PSO) [2], differential evolution (DE) [3], ant colony optimization [4], artificial bee colony algorithm [5], and firefly algorithm [6], form the hot topics in this area.

Cuckoo search algorithm (CSA), another adoption of biomimicry in the optimization technique which reproduces the breeding strategy of the best known brood parasitic bird, the cuckoos, has been proposed by Yang and Deb recently [7, 8]. Cuckoos, probably one of the most vicious and cunning species of all bird breeds, clandestinely lay their eggs in the nests of other host birds, sparing themselves the parental responsibilities of raising the young. In fact, cuckoos practice the art of deception all the time in their reproductive life. They mimic the colour and pattern of the host eggshell in order to disguise their eggs from being detected by the host birds. To make more space and food for their young chick, cuckoos will steal the host egg while sneaking their own into the nest. However, the relationship between the host species and the cuckoos is often a continuous arms race. The hosts learn to discern the imposters and they either throw out the parasitic eggs or desert the nest; the parasites improve the forgery skill to make their eggs to appear more alike with the host eggs.

The feasibility of applying the CSA to locate the global optimum for the optimization problems has been investigated in the literature. In the pioneering work of Yang and Deb, the CSA has been implemented successfully in optimizing several benchmark functions, and their findings showed that the global search ability of the CSA is more efficient than GA and PSO [7, 8]. On the other hand, the CSA has been employed in diverse domains since its inception; including engineering design process [9–12], chaotic system [13], wireless sensor networks [14, 15], structural optimization problem [9, 16, 17], image processing [18, 19], milling process [20], and scheduling problem [21–23]. Undoubtedly, its popularity increases unceasingly in the not-to-distant future.

Nevertheless, in real world situations, obtaining the exact global optimum is impracticable, as the underlying problems are always subjected to various uncertainties and constraints. In this case, instead of finding the actual optimum, the core consideration in selecting an appropriate optimization technique is how much improvement is achievable for a given application at a plausible computational complexity, with an acceptable error. The main thrust of this paper is therefore geared towards a modified CSA, which integrates an accelerated searching strategy in its computation. The improvement over the CSA is tested and validated through the optimization of several benchmarks. The paper is organized as follows. In Section 2, the standard CSA is introduced and its deficiencies are discussed. The modified CSA, specifically, the adaptive cuckoo search algorithm (ASCA), is proposed in Section 3, and the comparative results in evaluating the benchmark optimization functions are presented in Section 4. Finally, some conclusions are drawn in Section 5.

## 2. Cuckoo Search Algorithm

The CSA, which draws inspiration from cuckoo's adaption to breeding and reproduction, is idealized with the assumptions as follows:

- each cuckoo lays one egg in a randomly selected host nest at a time, where the egg represents the possible solution for the problem under study;
- the CSA follows the survival of the fittest principle. Only the fittest among all the host nests with high quality eggs will be passed on to the next generation;
- the number of host nests in the CSA is fixed beforehand. The host bird spots the intruder egg with a probability *p*_*a*_ ∈ [0,1]. For such incidents, the host bird will either evict the parasitic egg or abandon the nest totally and seek for a new site to rebuild the nest.

Derived from these assumptions, the steps involved in the computation of the standard CSA are presented in Algorithm 1 [7].

Such idealized assumptions in the CSA, similarly to what was previously proposed in other metaheuristic optimization approaches, make use of the ideas of elitism, intensification, and diversification. Start with a population of possible solutions, a new and potentially better solution (cuckoo egg) is generated. If the fitness value of this cuckoo egg is higher than another randomly selected egg from the available host nests, then it will replace that poor solution.

The cuckoo lays an egg at random location via Lévy flight, which is characterized by

(1) xi=(iter+1)=xi(iter)+α×lévy(λ),lévy(λ)=|Γ(1+λ)×sin⁡(πλ/2)Γ((1+λ)/2)×λ×2((λ−1)/2)|1/λ,

where *x*_*i*_ is the possible solution,* iter* denotes the current generation number, Γ is the gamma function which is defined by the integral: Γ(*x*) = ∫_0_^*∞*^ *e* ^−*t*^ *t* ^*x*−1^ *dt*, and *λ* is a constant (1 < *λ* ≤ 3).

The Lévy flight process is a random walk that forms a series of instantaneous jumps chosen from a heavy-tailed probability density function [24]. The step size *α*, which controls the scale of this random search patterns, helps exploit the search space around the current best solution and meanwhile explore the search space more thoroughly by far field randomization such that the search process can effectively move away from a local optima. Therefore, the value for *α* must be assigned judiciously. The search process will be less efficient if α is chosen as a small value, since the location for the new generated solution is near to the previous. On the other hand, if the value for *α* is too big, the new cuckoo egg might be placed outside the bounds. To balance the effectiveness for both intensification and diversification, Yang and Deb assigned the value of *α* as 1 [7].

Yang and Deb have also pointed out that the CSA outperforms the GA and the PSO in terms of the number of parameters to be adjusted [7]. In the CSA, only the probability of the abandoned nests *p*_*a*_ is tuned. However, the setting of *p*_*a*_ = 0.25 is sufficient enough, as they found out that the convergence rate of CSA is insensitive to *p*_*a*_. Thus, the fraction of nests to desert *p*_*a*_ is assigned as 0.25 in this study.

## 3. Adaptive Cuckoo Search Algorithm

For any optimization approach, finding the optimum solutions competently and accurately relies utterly on the inherent search process. The effectiveness of the standard CSA is unquestionable, meaning that when given enough computation time, it is guaranteed to converge to the optimum solutions eventually. However, the search process may be time consuming, due to the associated random walk behavior [24]. In order to improve the convergence rate while maintaining the eye-catching characteristics of the CSA, an accelerated searching process which, similarly to the inertia weight control strategy in the PSO [25], is proposed here.

The step size *α*, which manages the local and global searching, is assigned as constant in the standard CSA, where *α* = 1 is applied. In this present work, a new adaptive cuckoo search algorithm (ACSA) is presented. Instead of using a constant value, the step size *α* is adjusted adaptively in the proposed ACSA, based on the assumption that the cuckoos lay their eggs at the area with a higher egg survival rate. In this regard, by adjusting the step size *α* adaptively, the cuckoos search around the current good solutions for laying an egg as this region probably will contain the optimal solutions, and, on the contrary, they explore more rigorously for a better environment if the current habitat is not suitable for breeding. The step size *α* is determined adaptively as follows:

(2) $$\begin{array}{c} \alpha =\{\begin{array}{cc} \alpha_{L}+\left(\alpha_{U}-\alpha_{L}\right)\frac{F_{j}-F_{\min}}{F_{\text{avg}}-F_{\min}}, & F_{j}\leq F_{\text{avg}}\\ \frac{\alpha_{U}}{\sqrt{t}}, & F_{j}>F_{\text{avg}}, \end{array} \end{array}$$

where *α*_*L*_ is the predefined minimum step size, *α*_*U*_ is the predefined maximum step size, *F*_*j*_ is the fitness value of the *j*th cuckoo egg, and *F*_min⁡_ and *F*_avg_ denote the minimum and the average fitness values of all host nests, respectively. The flow of the ACSA is given in Algorithm 2.

The step size *α* determines how far a new cuckoo egg is located from the current host nest. Specifying the minimum and the maximum Lévy flight step size values properly is crucial such that the search process in the ACSA is neither too aggressive nor too ineffective. The *α*_*L*_ and *α*_*M*_ are chosen based on the domain of **x**_*i*_. Additionally, as precautionary measure, if the ACSA generates a cuckoo egg that falls outside the domain of interest, its position will remain unchanged.

## 4. Numerical Simulations

To evaluate the feasibility of the proposed ACSA, the algorithm is applied to optimize the five benchmark functions with known global optima, where two of which are unimodal and three of which are multimodal. The optimization performance is compared with the standard CSA. For each test function, the initial populations of 20 host nests are generated randomly. The simulations are performed for 30 independent runs. The optimization process stops if the best fitness value is less than a given tolerance *ξ* ≤ 10^−5^. For both the CSA and ACSA, the Euclidean distance from the known global minimum to the location of the best host nest with the lowest fitness value is evaluated in each iteration. The average of the distance difference for each loop from all the 30 trials is then measured.

In addition, to authenticate the statistical significance of the proposed ACSA, the two-tailed *t*-test is applied. The null hypothesis is rejected at the confidence interval of 5% level, if the difference of the means of both CSA and ACSA is statistically significant. The results are presented in the last column of Table 1.

### 4.1. Ackley's Function

The Ackley's function is a multimodal function which is described as [26]

**Figure 1:**
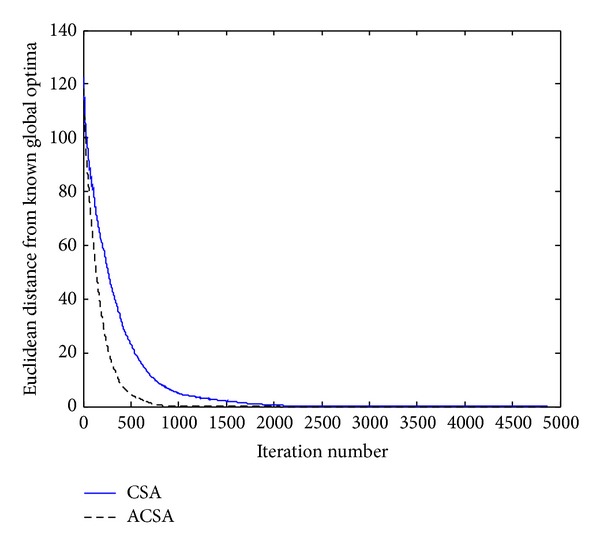
Comparison of the performance of the standard CSA and the proposed ACSA for the Ackley's function.

(3) f(x)=−20exp⁡⁡(−0.21d∑i=1dxi2) −exp⁡(1d∑i=1dcos⁡⁡(2πxi))+20+e,

where *x*_*i*_ ∈ [−32.768,32.768] and data dimension, *d* = 50, has a global minima of *f*_∗_ = 0, at *x*_∗_ = (0,0,…, 0). The *α*_*L*_ and *α*_*M*_ are chosen as 0.2∗domain *x*_*i*_ and 0.8∗domain *x*_*i*_, respectively.  Figure 1 compares the relative performances of the CSA and ACSA in optimizing Ackley's function. As observed in this figure, apparently the convergence rate of the ACSA is better than the standard CSA on this test function. Considering Table 1 which summarizes the average cycles needed for both the algorithms to meet the stopping criterion, it can be clearly seen that the CSA takes more iteration to converge; however, the proposed ACSA reaches the global optima about one time faster on average. The ACSA is able to find the global solution, approximately after 2500 fitness function evaluations, as opposed to CSA which reaches the global solution after 4000 objective function evaluations.

### 4.2. De Jong's Function

**Figure 2:**
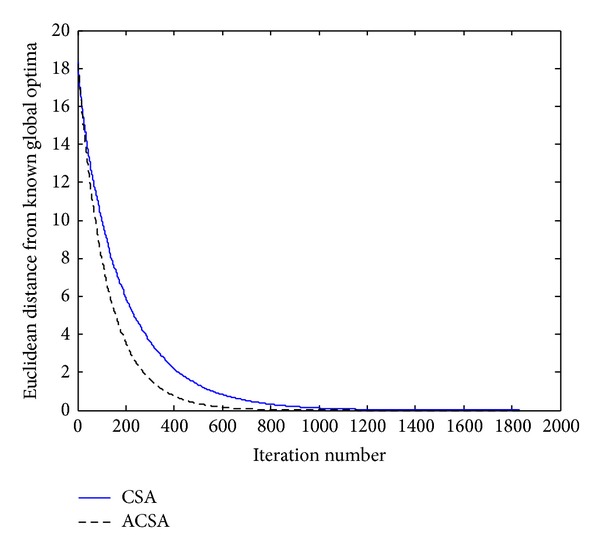
Comparison of the performance of the standard CSA and the proposed ACSA for the de Jong's function.

As the simplest unimodal test function, the de Jong's function is given by [27]

(4) $$\begin{array}{c} f\left(x\right)=\overset{d}{\underset{i=1}{\sum }}x_{i}^{2}, \end{array}$$

where *x*_*i*_ ∈ [−5.12,5.12] and *d* = 50. This is a sphere function, with the known global minimum of *f*_∗_ = 0, at *x*_∗_ = (0,0,…, 0). The *α*_*L*_ and *α*_*M*_ are chosen as 0.2∗domain *x*_*i*_ and 0.8∗domain *x*_*i*_, respectively. The results achieved by the standard CSA and the proposed ACSA in optimizing the de Jong's function are presented in Figure 2. Due to the simplicity of the test function, it can be found that both algorithms take considerably less iteration steps for convergence but, the ACSA with adjustable step size is much more efficient than the standard CSA. As shown in Table 1, the ACSA reaches the known global optimum in a mean of 1000 cycles, while the CSA requires a longer processing time in order to converge.

### 4.3. Griewank's Function

**Figure 3:**
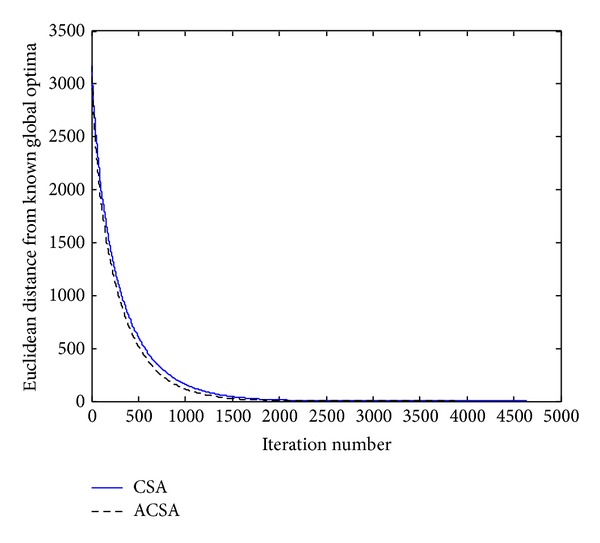
Comparison of the performance of the standard CSA and the proposed ACSA for the Griewank's function.

Figure 3 depicts the optimization results of the CSA and ACSA in terms of convergence characteristics for the multimodal Griewank's function [28]:

(5) $$\begin{array}{c} f\left(x\right)=\frac{1}{4000}\overset{d}{\underset{i=1}{\sum }}x_{i}^{2}-\overset{d}{\underset{i=1}{\prod }}\cos\left(\frac{x_{i}}{\sqrt{i}}\right)+1, \end{array}$$

where *x*_*i*_ ∈ [−600,600] with *d* = 100. This is a high dimensional multimodal function, with the known global minimum of *f*_∗_ = 0 at *x*_∗_ = (0,0,…, 0). The *α*_*L*_ and *α*_*M*_ are chosen as 0.02∗domain *x*_*i*_ and 0.08∗domain *x*_*i*_, respectively. Considering Figure 3 and Table 1, the obtained results suggest that the ACSA shows improved convergence efficiency. Moreover, it can be inferred from Table 1 that the superiority of the ACSA is obvious, as it outperforms the CSA in terms of the best, worst, average, and standard deviation, for the number of iterations needed in order to reach the known global optimum.

### 4.4. Rastrigin's Function

**Figure 4:**
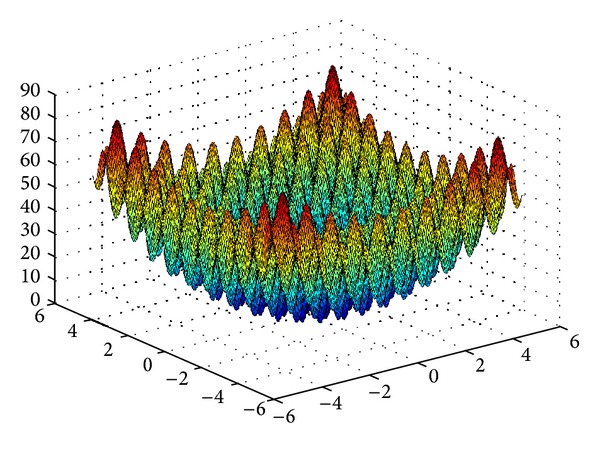
The 3-dimensional surface plot for the Rastrigin's function.

The multimodal Rastrigin's function is defined as [29]

(6) $$\begin{array}{c} f\left(x\right)=10d+\overset{d}{\underset{i=1}{\sum }}\left[x_{i}^{2}-10\cos\left(2\pi x_{i}\right)\right], \end{array}$$

where *x*_*i*_ ∈ [−5.12,5.12] and *d* = 100, with a global minimum of *f*_∗_ = 0 at *x*_∗_ = (0,0,…, 0). The *α*_*L*_ and *α*_*M*_ are chosen as 0.2∗domain *x*_*i*_ and 0.8∗domain *x*_*i*_, respectively. Getting the global minimum of the Rastrigin's function is a difficult process, as this test function has many local minima, which can be observed in its 3-dimensional surface plot in Figure 4. A more challenging 100-dimensional Rastrigin's function is chosen in this case to corroborate the global searching ability of the proposed algorithm.

**Figure 5:**
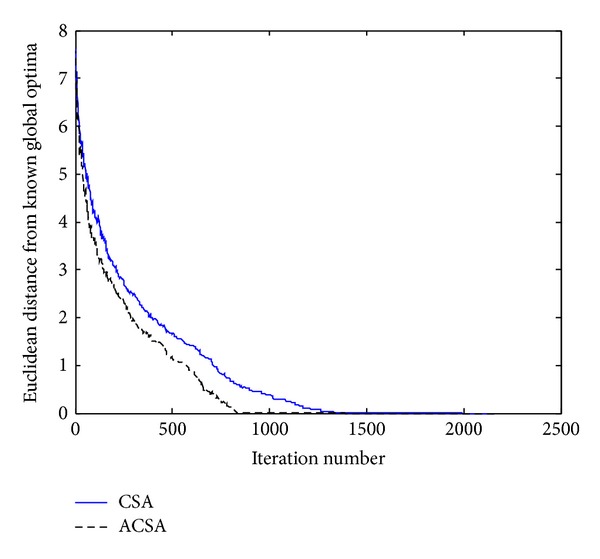
Comparison of the performance of the standard CSA and the proposed ACSA for the Rastrigin's function.

Figure 5 presents the optimization performances of the CSA and ACSA for Rastrigin's function. It can be deduced from the figure that the proposed ACSA is superior to the standard CSA in terms of the convergence rate. It is evident that initially, the CSA appears to converge faster to the known global minimum than ASCA. However, as the evolution continues, the ACSA tends to get closer to the true solution than CSA. This is presumably due to the CSA is getting trapped in local solution, as there are many local minima present in this test function. Moreover, the optimization process of CSA may span a longer period of time than the proposed ACSA as it makes too small and cautious steps while exploring the search space, that is, the *α* which controls the scale of the search patterns is specified as 1. On the contrary, the ACSA with adaptive step size control strategy performs more rigorous search through the solution space. The ACSA is able to find the global minimum in a mean of 20000 iterations. While on average, the CSA needs 2000 more iterations in order to reach the optimal solution.

### 4.5. Rosenbrock's Function

**Figure 6:**
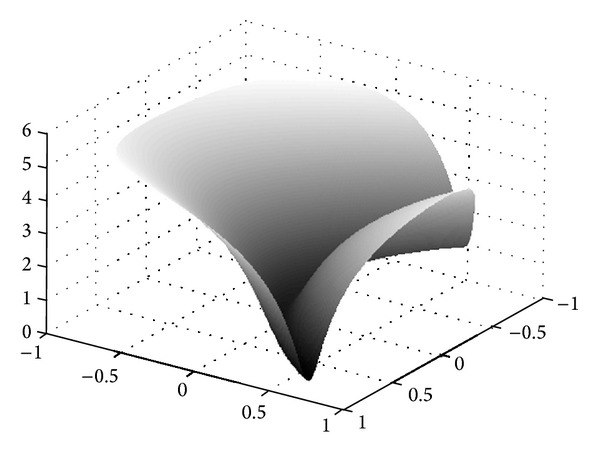
The 3-dimensional surface plot for the Rosenbrock's function.

Figure 6 illustrates the 3-dimensional surface plot for the Rosenbrock's function, which is defined as [30]:

(7) $$\begin{array}{c} f\left(x\right)=\overset{d-1}{\underset{i=1}{\sum }}\left[{\left(1-x_{i}\right)}^{2}+100{\left(x_{i+1}-x_{i}^{2}\right)}^{2}\right], \end{array}$$

where *x*_*i*_ ∈ [−100,100] and *d* = 10. This test function has a global minimum of *f*_∗_ = 0 at *x*_∗_ = (1,1,…, 1). The *α*_*L*_ and *α*_*M*_ are chosen as 0.02∗domain *x*_*i*_ and 0.08∗domain *x*_*i*_, respectively. As shown in this figure, there is a long, narrow and parabolic shaped valley in the surface plot. This is the region where the global minimum is resided. Although finding this valley is not tedious, reaching the global optima is difficult [24].

**Figure 7:**
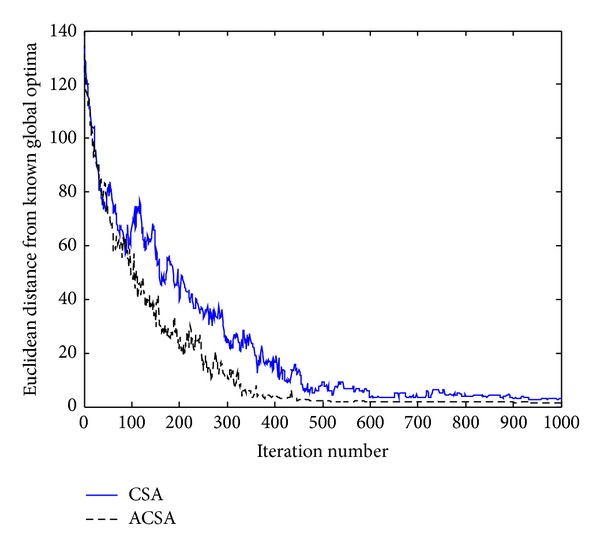
Comparison of the performance of the standard CSA and the proposed ACSA for the Rosenbrock's function.

The performances of the CSA and ACSA in terms of the convergence efficiency are shown in Figure 7. Comparing the best obtained results in among all the 30 trials, apparently that the proposed ACSA speeds up the computation more than one time faster, as compared to the standard CSA. Moreover, it can be noted that the average number of evaluations needed by the CSA is much inferior to the longest iteration achieved by the ACSA from among all the 30 independent runs.

### 4.6. Performance Comparison with Other Optimization Algorithms

In comparing the performances of different algorithms in optimizing the same benchmark functions, several ways with different stopping criteria can be considered, in which comparison of best fitness value for a fixed number of fitness function evaluations, a commonly used approach, has been adopted in this study. The other optimization algorithms considered are DE, evolutionary programming (EP), GA, PSO, simulated annealing (SA), and CSA. The simulations of all algorithms are performed for 30 independent runs with the number of fitness function evaluations is set to 2000. The best fitness value in each iteration is evaluated, and its average at each iteration from all the 30 trials is the measured. The obtained average best fitness values at fixed iteration number of 1, 500, 1000, 1500, and 2000 in optimizing the benchmark functions are summarized in Table 2.

As shown in this table, the proposed ACSA has higher precision than any of the other algorithms for all benchmarks considered, except for Griewank's function. The DE produces solution that is closer to the global optima than the proposed ACSA in this case. However, it is pertinent to note that there is no or marginal improvement in DE after 500 function evaluations. The obtained best fitness value only decreases slightly from 0.35 to 0.33, which might indicates that the DE get trapped in local optima. In fact, as shown in Table 2, the DE, EP, and PSO algorithms usually converge faster initially, but they often get stuck in local optima easily, which is particularly obvious in the case of Ackley, de Jong, and Griewank's functions. On the other hand, the proposed ACSA is getting closer to the global optima as the iteration increases gradually. The superiority of ACSA is more noticeable in the case of Ackley, de Jong, and Rastrigin's function, where the ACSA reaches the near-optimal solution after 1500 function evaluations.

## 5. Conclusions

In this paper, by scrutinizing the advantages and the limitations of the standard CSA, a new modified CSA, specifically the ACSA, which adopts an adjustable step size in its computation, has been proposed. From all the considered benchmark optimization functions, the superiority of the ACSA over the standard CSA is validated, in terms of the convergence characteristics. The proposed algorithm preserves the uniqueness and the fascinating features of the original CSA, as both of the algorithms are able to find the global optimum when given enough computation time but the ACSA is able to converge faster in less iteration. This is probably due to its capability to concurrently refine the local search phase around the current good solutions while exploring more aggressively for the optimal solutions, attributed to the adopted adaptive step size. It is worth mentioning that through empirical simulations, increasing the maximum step size value will encourage a more thorough global exploration and eventually lead to faster convergence; however, the generated new solutions might fall outside the design domain for some cases. Thus, a moderate value is chosen in this study.

## Figures and Tables

**Algorithm 1:**
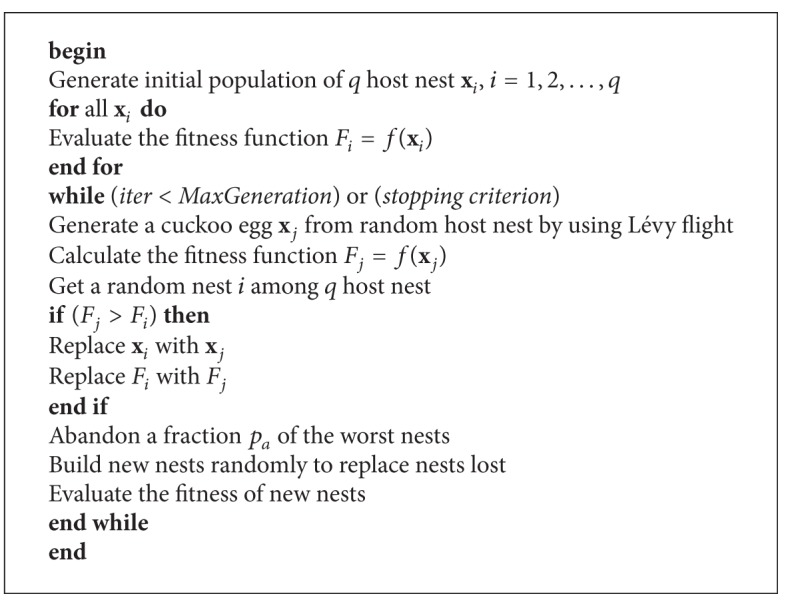
Cuckoo search algorithm.

**Algorithm 2:**
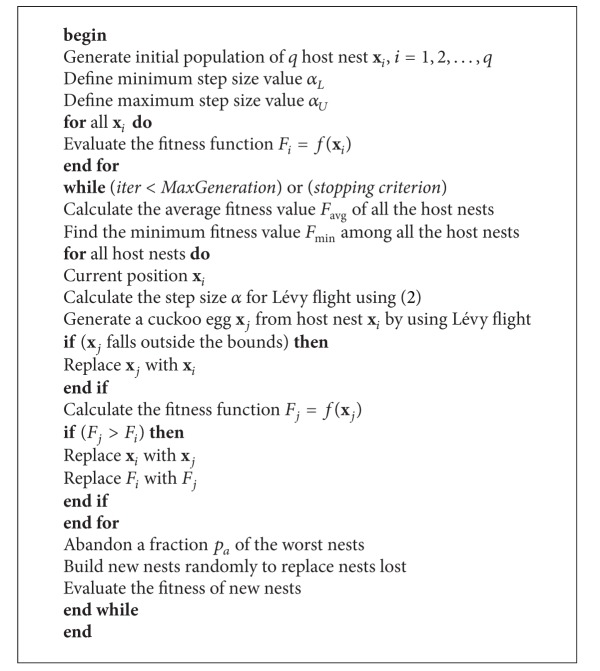
Adaptive cuckoo search algorithm.

**Table 1:** Performance comparison of ACSA with CSA (in terms of the number of iterations needed in order to converge).

Benchmark function	CSA				ACSA				Significant
	Best	Worst	Mean	SD	Best	Worst	Mean	SD	
Ackley	3884	4862	4222	250.74	1912	2068	1989	41.03	Yes
De Jong	1690	1833	1763	37.55	1087	1158	1121	19.81	Yes
Griewank	4287	4632	4499	75.63	3617	3880	3725	63.77	Yes
Rastrigin	1582	1994	1734	118.73	1190	1389	1286	50.69	Yes
Rosenbrock	17519	33929	25402	4592.24	9131	28803	22164	5416.74	Yes

*SD denotes the standard deviation.

**Table 2:** Performance comparison of ACSA with other optimization methods (in terms of fixed iteration number).

Benchmark function	Generation	Average best fitness value over 30 independent runs						
		DE	EP	GA	PSO	SA	CSA	ACSA
Ackley	1	19.15	19.62	20.93	20.77	21.08	20.95	20.90
	500	2.68	4.94	11.54	2.69	20.54	11.16	3.76
	1000	2.68	4.63	8.44	2.69	19.43	4.01	0.06
	1500	2.68	4.41	6.94	2.69	17.57	1.90	6.39*E* − 04
	2000	2.68	4.29	6.00	2.69	15.39	0.50	8.71*E* − 06

de Jong	1	32.88	41.85	334.35	334.12	332.88	335.59	343.79
	500	0.02	1.44	17.79	0.27	89.98	1.91	0.14
	1000	0.02	1.21	7.65	0.27	68.02	0.02	7.58*E* − 05
	1500	0.02	1.13	4.52	0.27	40.57	1.29*E* − 04	3.91*E* − 08
	2000	0.02	1.04	2.96	0.27	20.32	1.06*E* − 06	2.10*E* − 11

Griewank	1	96.76	155.82	2521.20	2477.39	289.59	2516.57	2543.72
	500	0.35	0.92	342.43	2.13	252.83	89.87	69.32
	1000	0.34	0.85	199.79	2.13	197.62	7.40	4.23
	1500	0.33	0.83	136.00	2.13	147.97	1.46	1.17
	2000	0.33	0.78	103.27	2.13	106.96	1.01	0.70

Rastrigin	1	119.64	125.49	122.83	126.52	191.39	123.00	122.26
	500	14.69	45.90	8.16	28.09	186.84	3.67	2.94
	1000	14.69	41.27	4.27	28.08	173.08	0.62	0.25
	1500	14.69	38.54	3.21	27.84	165.48	1.16*E* − 03	8.82*E* − 05
	2000	14.69	36.82	2.28	27.78	158.28	9.72*E* − 07	8.65*E* − 09

Rosenbrock	1	3.11*E* + 09	5.10*E* + 09	4.81*E* + 09	5.54*E* + 09	1.79*E* + 10	5.44*E* + 09	5.90*E* + 09
	500	1.09*E* + 05	3.39*E* + 04	3.28*E* + 05	7.34*E* + 04	7.93*E* + 09	205.31	61.01
	1000	1.04*E* + 05	1.31*E* + 04	3.79*E* + 04	7.31*E* + 04	4.30*E* + 08	13.82	6.37
	1500	1.04*E* + 05	9.34*E* + 03	8.99*E* + 03	7.31*E* + 04	3.02*E* + 05	4.72	3.17
	2000	1.04*E* + 05	8.36*E* + 03	5.21*E* + 03	7.31*E* + 04	8.97*E* + 04	2.94	1.75
